# High-Pressure Phases of SnO and PbO: A Density Functional Theory Combined with an Evolutionary Algorithm Approach

**DOI:** 10.3390/ma14216552

**Published:** 2021-11-01

**Authors:** Long Truong Nguyen, Guy Makov

**Affiliations:** Department of Materials Engineering, Faculty of Engineering Sciences, Ben-Gurion University of the Negev, Beer-Sheva 84105, Israel; nguyenl@post.bgu.ac.il

**Keywords:** phase transition, high-pressure, density-functional theory, evolution algorithm

## Abstract

Tin monoxide, SnO, and its analog, lead monoxide, PbO, have the same tetragonal *P4/nmm* structure, shaped by nonbonding dispersion forces and lone pairs. The high-pressure phases of SnO and PbO have been explored in several experimental and theoretical studies, with conflicting results. In this study, the high-pressure structures of SnO and PbO are investigated using density functional theory calculations combined with an evolutionary algorithm to identify novel high-pressure phases. We propose that the monoclinic *P2_1_/m* SnO and orthorhombic *Pmmn* PbO phases, which are metastable at 0 GPa, are a slight rearrangement of the tetragonal *P4/nmm*-layered structure. These orthorhombic (and their closely related monoclinic) phases become more favored than the tetragonal phase upon compression. In particular, the transition pressures to the orthorhombic γ-phase *Pmn2_1_* of SnO/PbO and the monoclinic phase *P2_1_/m* of SnO are found to be consistent with experimental studies. Two new high-pressure SnO/PbO polymorphs are predicted: the orthorhombic *Pbcm* phase of SnO and the monoclinic *C_2_/m* of PbO. These phases are stabilized in our calculations when P > 65 GPa and P > 50 GPa, respectively. The weakening of the lone pair localization and elastic instability are the main drivers of pressure-induced phase transitions. Modulations of the SnO/PbO electronic structure due to structural transitions upon compression are also discussed.

## 1. Introduction

SnO and PbO are a group IV metal oxide semiconductors, which serve as functional materials in a wide variety of applications. SnO has emerged as a candidate for p-type thin-film transistor as a transparent p-type semiconductor with high hole mobility and a small indirect bandgap (0.7 eV) [1,2,3]. By contrast, PbO is an n-type semiconductor with a wider indirect bandgap (1.9 eV) and with applications that include serving as a photoconductive material in imaging devices and X-ray detectors [4,5,6] as well as an anode in lead–acid and lithium–ion batteries [7,8].

Stable α-phase SnO and PbO have a *P4/nmm* tetragonal structure. Under ambient conditions, PbO can also be obtained in the metastable orthorhombic *Pbcm* β-phase. An irreversible α–β transition in PbO occurs at 540 °C, but not in SnO. Instead, at high temperatures, SnO decomposes in a disproportionation reaction to Sn and SnO_2_ at temperatures greater than 300 °C. The tetragonal *P4/nmm* structure consists of double-layered Sn/Pb atoms stacked along the c axis, which sandwich a single O layer. The Sn/Pb atoms are positioned at the apex of tetrahedrally square-based O atom pyramids [9,10]. This square pyramidal *P4/nmm* arrangement can be considered a distortion of the cubic CsCl structure by elongating the c axis. One particular atomic characteristic of SnO and PbO is the formation of stereochemically active lone pairs by overlapping metal s states with the oxygen 2p states, resulting in bonding and antibonding combinations. The bonding combination contains mainly an oxygen 2p state, whereas the metal s and p states together with the oxygen 2p state comprise the antibonding combination. The stereochemically active lone pairs are considered responsible for directing the Sn/Pb-O layer into a tetragonal or orthorhombic structure [9,10].

In recent years, the search for SnO and PbO phase transitions at high pressures has been the object of theoretical and experimental studies [11,12,13,14,15]. A second-order pressure-induced tetragonal-to-orthorhombic (α → γ) transition for both SnO and PbO was proposed by Adams et al. [11] at P = 2.5 GPa and 0.7 Gpa, respectively. The γ-phase was assumed to be an orthorhombic phase with either the *Pmmn*, *P2_1_mn*, or *Pmn2_1_* space group. A further increase in pressure induced a structural transition to the β-phase (*Pbcm*) in PbO, whereas transformation into this *Pbcm* structure of SnO was not found [11]. Subsequent hydrostatic compression experiments up to P = 50 GPa have not demonstrated the splitting of lattice parameters *a* and *b* from tetragonal to orthorhombic SnO [12,15]. Therefore, Giefers et al. [12,16,17] and Wang et al. [15] have argued that the observation of the γ-phase in SnO and PbO is the result of shear stress applied during the experiment rather than an intrinsic property of hydrostatic compression. In addition, using different pressure-transmitting media in the compression experiments led to the identification of a monoclinic *P2_1_m* phase of SnO from a diffraction pattern at 17.5 GPa [15]. The deviation of the unit cell angle from a tetragonal structure (and splitting of lattice parameters *a* and *b*) implies a slight shift of adjacent layers at high pressure. The possibility of a second-order transition of SnO from *P4/nmm* to γ-phase *Pmn2_1_* in the range of 0–5 GPa was evaluated using density functional theory (DFT) calculations [14]. The authors suggested that the softening of the B_1g_ mode in compressed tetragonal SnO drove this transition. Unfortunately, this might not be in agreement with experimental results from [11], as the Raman spectra did not verify the softening of the B_1g_ mode in the PbO α → γ transition. Hence, a more detailed examination of the structural variation and phonon dispersion in compressed SnO and PbO is required.

In addition, SnO undergoes a pressure-induced transition at approximately 5 GPa, from semiconductor to semi-metallic, accompanied by a sudden change in electrical conductivity [18]. Several DFT calculations have indicated that this transition is the consequence of closing the Sn–Sn interlayer distance under hydrostatic conditions [9,13,19]. Christensen et al. estimated that the indirect bandgap depends on the Sn–Sn interlayer distance using the formula: $dE_{bandgap}/d\left[\ln\left(c/a\right)\right]=21\;\mathrm{eV}$ [9]. Therefore, an estimate of the bandgap closure pressure was obtained by replacing the underestimated bandgap from DFT calculations with an experimental bandgap, which predicted the bandgap closing at P = 4.8 GPa and c/a = 1.2252. Based on a DFT calculation, McLeod et al. [19] suggested that the tail of hybridized oxygen 2p states, with tin 5s/5p states in the lone pairs region, filled the semiconducting bandgap due to the increase in the Sn–Sn interaction between two layers. The hybridized states of oxygen 2p with tin 5s/5p were also distorted into hole/electron pockets upon compression. This tendency induced superconductivity in SnO in the range of 5–20 GPa [19]. The phase diagram of SnO superconductivity under pressure, with a maximum T_c_ = 1.5 K at p ~ 8.8 GPa, was reported by Chen et al. [13]. In contrast, the pressure effect on the PbO electronic structure has drawn less attention than its SnO analog due to the wider indirect bandgap of PbO. DFT calculations predicted that the PbO indirect bandgap decreases during compression (as $dE_{bandgap}/dP=-0.16\;\mathrm{eV}/\mathrm{GPa}$), whereas the direct bandgap increases with a rate of $dE_{bandgap}/dP=0.13\;\mathrm{eV}/\mathrm{GPa}$ [20].

Although investigated in several studies, the pressure-induced transitions of SnO and PbO remain unresolved due to the many questions arising regarding their structural transitions upon compression. This first-principles DFT study examines the effect of pressure on the lattice structure, electronic structure, and phonon dispersion for high-pressure and ambient-phase SnO compared to PbO. Candidate high-pressure phases are identified via an evolutionary algorithm approach. One potential problem in using an evolutionary algorithm for structure prediction at high pressure is that choosing a single pressure value for the search could miss phases stable only in limited pressure ranges. Hence, our study is conducted at ambient conditions and several high-pressure points to discover new stable phases. The high-pressure phase-transition mechanism is discussed in the context of the lone pairs and the elastic properties.

## 2. Calculation Methods

The DFT calculations were performed with the Quantum Espresso package [21] using plane-wave norm-conserving pseudopotentials [22]. The exchange correlation was represented in the generalized gradient approximation by the Perdew–Burke–Ernzerhof functional [23]. Using a variable cell relaxation procedure implemented in Quantum Espresso, the structure of tetragonal SnO and PbO under hydrostatic compression was optimized in the range of 0–100 GPa for SnO and 0–50 GPa for PbO. The initial input for this procedure was based on experimental equilibrium lattice constants and atomic positions. These optimized tetragonal structures of SnO and PbO serve as reference states for comparison with alternative phases formed upon hydrostatic compression by examining their structural, electronic, and dynamical properties. This structural optimization was performed with a high energy cutoff at 1224 eV and dense 12 × 12 × 8 k-point grids. Self-consistent total energy and a relaxed force threshold converged to better than the 10^−12^ eV/unit cell and 0.025 eV/Å, respectively. These choices ensure high accuracy in determining the lattice constants, which has been estimated to have less than 10% probability of errors greater than 0.2% [24].

A wide-ranging investigation using an evolutionary algorithm was then undertaken to predict the low enthalpy structures at selected pressures. We employed a genetic algorithm (GA) search-based optimization technique based on an analogy with genetics and natural selection principles and implemented in the GA code XtalOpt [25]. The principle of applying GA to phase prediction is to create a large population of possible phases and calculate their enthalpy using DFT methods. The lower enthalpy species are then reserved for producing the next generation via operations that mutate the parent’s structure: applying a strain or ripple or exchanging the atomic positions, followed by a crossover between two parental phases [25]. We employ GA global optimization to find the low enthalpy phases until the best structure remains unchanged for the next three generations. To reduce the computational cost of the global search, an energy cutoff at 544 eV was employed, and self-consistent calculations were performed using 2 × 2 × 2 k-point grids. The self-consistent energy converged to better than the 10^−6^ eV/unit cell. All calculations of structural relaxation were performed using the Broyden–Fletcher–Goldfarb–Shanno algorithm. The atomic structure was determined by allowing the positions and unit-cell parameters to relax until forces were less than 0.025 eV/Å and the stress less than 0.5 kbar. We performed the global optimization of GA at several high-pressure points to avoid missing intermediate phase transitions. SnO was explored at 0, 5, 20, 50, and 100 GPa, and PbO was studied at 0, 1, 5, 20, and 50 GPa. After identifying the metastable phases with the GA search, a refinement at the specific investigated pressure was carried out using the same variable cell relaxation conditions of 1224 eV cutoff energy and a dense k-point grid, as in the optimization for the reference tetragonal structure. This procedure ensures accuracy and convergence when comparing the lattice structure and enthalpies upon compression between the GA phases and reference tetragonal SnO/PbO.

The dynamical stability of the candidate phases under compression was evaluated by calculating the phonon dispersion curves. Phonon calculations were performed using density functional perturbation theory [26] with grids of 6 × 6 × 6 k-points and 3 × 3 × 3 q-points. The band structure and the density of state (DOS) were calculated for the final refined structures. Crystal orbital Hamilton population (COHP) calculations [27] were employed to interpret the bonding and antibonding characteristics.

## 3. Results

### 3.1. Genetic Algorithm Prediction of the SnO/PbO Phase at 0 GPa

#### 3.1.1. SnO Metastable Phase at 0 GPa

From the GA and the DFT calculations at 0 GPa, we obtained the thermodynamically stable phase to be tetragonal *P4/nmm*, identical to experimental observations [11,12,16]. We also identified by the GA additional metastable phases. These metastable phases were ordered by their formation energy relative to the reference tetragonal phase. The formation energy is given by the difference between the total energy, *E_GA_*, of the metastable phase and that of the tetragonal phase, *E_tetra_*, at 0 GPa:

*E*_*f*_ = *E*_*GA*_ − *E*_*tetra*_ (1)

The metastable phases of SnO at 0 GPa, which the GA identified, are presented in Table 1 (in the order of their formation energies) with their phase structures. The first two phases (*Pmmn* and *P2_1_m*) are distortions of the square pyramidal shape of ambient *P4/nmm* into trigonal bipyramidal structures. The *Pbcm* phase contains a more distorted Sn–O pyramid shape and a disordered trigonal bipyramidal formation. The *P2_1_/c* and *P2_1_3* phases form tetrahedral structures, similar to the SnS *Pnma* and π-cubic phases [28]. The last phase considered in Table 1 has the same space group as the ground state reference structure—tetragonal *P4/nmm*—but presented as an octahedral arrangement. Each Sn atom is bonded to five O atoms so that one more Sn–O bond is added to the original square pyramid. It should be noted that our predicted *P2_1_/c* structure is similar to that found in Ref. [29] and has the same formation energy *E_f_* of 0.06 eV.

**Figure 1:**
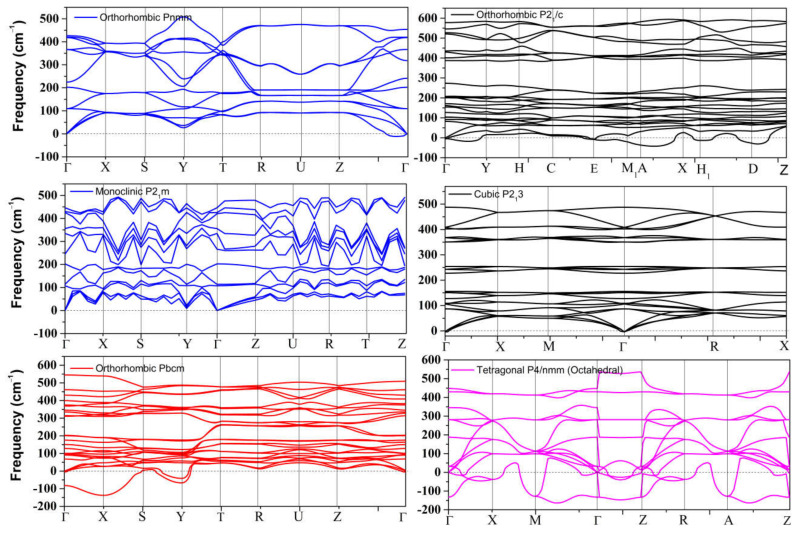
Phonon dispersion of all metastable phases for SnO at 0 GPa.

The dynamical stability of the proposed metastable phases was examined by calculating the phonon dispersion curves for each phase, the results of which are presented in Figure 1. These results show that only the distorted monoclinic phase *P2_1_m* can be stabilized at 0 GPa. The other metastable phases of SnO are not dynamically stable. The *Pmmn* and *P2_1_3* phonon spectra both contain a small range of optical branches near the Γ point with imaginary frequencies. The tetrahedral *P2_1_/c* contains several imaginary frequencies, indicating an unstable structure at 0 GPa. The structures of *Pbcm* and octahedral *P4/nmm* are also unstable, but these two phases can be stabilized at higher pressure, as we report in Section 3.2.2.

#### 3.1.2. PbO Metastable Phase at 0 GPa

**Figure 2:**
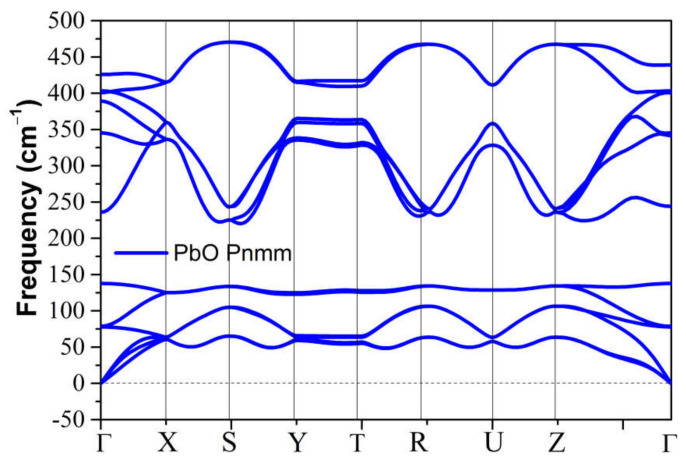
Phonon dispersion of the orthorhombic *Pmmn* phases for PbO at 0 GPa.

The metastable phases of PbO at 0 GPa, which the GA identified, are presented in Table 2 in the order of their formation energies. The formation energy of the trigonal bipyramidal *Pmmn* PbO phase is very close to the tetragonal ground-state phase. It is stable at 0 GPa as no imaginary (negative) phonon frequencies were found in the phonon dispersion relations (Figure 2). The splitting ratio of the lattice parameters *a* and *b* in the orthorhombic *Pmmn* structure is 5%. This splitting coincides with the orthorhombic PbO phase found experimentally close to 0 GPa [16]. The octahedral structures in the *Pmn2_1_* and *P42_1_m* phases are less favorable in energy than the trigonal bipyramidal *Pmmn* phase. Our phonon calculations also indicate that these octahedral phases are dynamically unstable. The *C_2_* phase is a distorted version of the tetrahedral structure *P2_1_c* found in SnO. The tetrahedral π-cubic *P2_1_3* and tetragonal *P4_2_* phases have higher formations energies. The *C_2_*, *P2_1_3*, and *P4_2_* phases formed by the tetrahedral bonding of PbO were all unstable at 0 GPa. Therefore, the only dynamically stable PbO and SnO structures at 0 GPa were the square pyramidal *P4nmm* phase and the trigonal bipyramidal *Pmmn* and *P2_1_/m* phases.

### 3.2. High Pressure Structures of SnO and PbO

#### 3.2.1. The Structural Variation of Tetragonal SnO and PbO upon Compression

**Figure 3:**
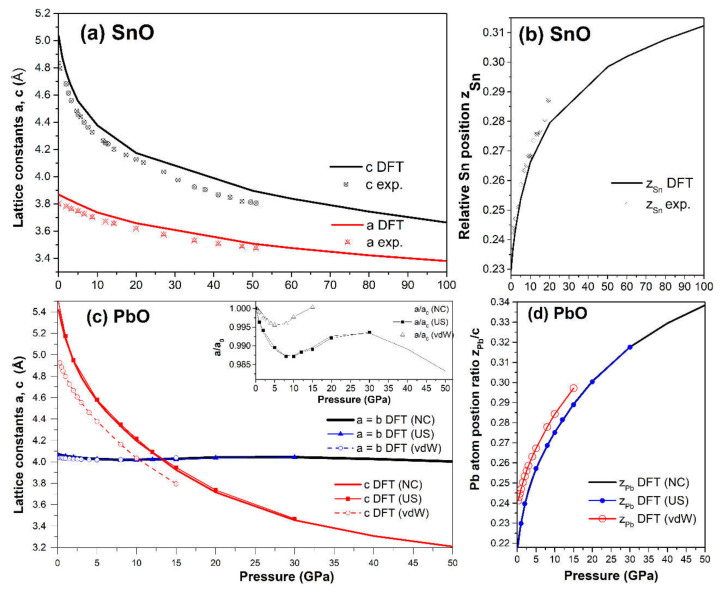
Calculated tetragonal SnO/PbO structural parameters: (**a**) Lattice parameters *a* and *c* vs. pressure of SnO compared with experimental measurements [15]; (**b**) Lattice parameters *a* and *c* vs. pressure of PbO. The calculation is validated with ultrasoft pseudopotential and vdW hybrid functionals vdw-ob-ft86 [30]. Inset: Enlarged view of the variation of parameter a in PbO; (**c**) Relative Sn atomic position z_Sn_ vs. pressure compared to experimental measurements [12]; (**d**) Relative Pb atomic position z_Pb_ vs. pressure.

We calculated the variation in the structural properties of tetragonal SnO and PbO upon compression, and the results are presented in Figure 3. In the 0–100 GPa range of SnO and 0–50 GPa in PbO, the *a*-lattice parameter showed lower compressibility than parameter *c,* i.e., the *c/a* ratio decreased. The gradual transformation of the tetragonal SnO lattice parameters agrees very well with experimental measurements in the pressure range of 0–50 GPa [12,15] (Figure 3a). A similar gradual decrease in the lattice parameters for *P4/nmm* PbO was observed in the pressure range of 0–4 GPa [16].

For PbO, the *c/a* ratio dropped below one at approximately 13 GPa. The PbO *a*-lattice parameter exhibited nonmonotonous pressure dependence; its value first decreased, then began to increase at 10 GPa, and finally decreased again at 30 GPa (see inset in Figure 3c). As the magnitude of this variation is less than 0.1Å, we confirmed this result by repeating the calculations with two alternatives to the main approximations: an ultrasoft pseudopotential and a vdW functional (vdw-ob-ft86) with results presented in the inset of Figure 3c. The inclusion of the van-der-Waals correction strongly affects the interaction between adjacent layers in SnO and PbO. Therefore, the calculation of c lattice parameters is shifted slightly in Figure 3c. No abrupt change was spotted in the trend of the Sn/Pb position in the unit cell with pressure (Figure 3b,d).

**Figure 4:**
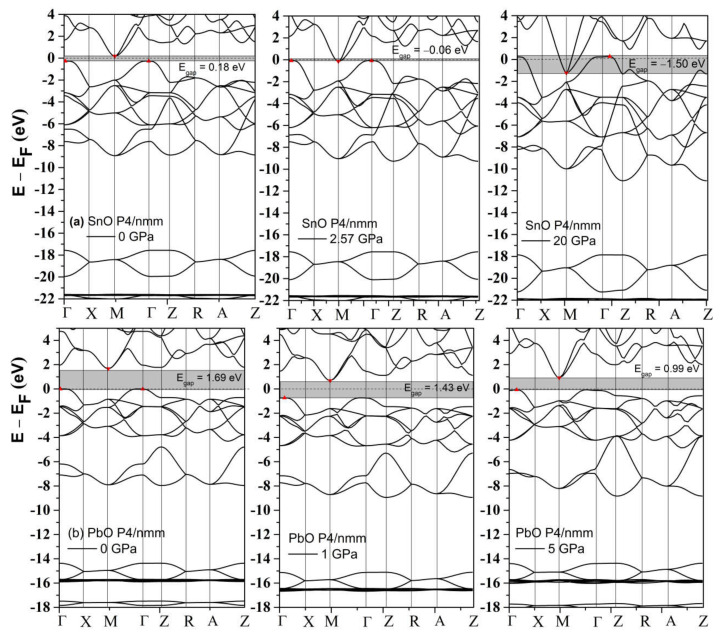
The calculated band structures of tetragonal SnO and PbO upon compression. The valence band maximum and conduction band minimum are marked as red triangle points.

The band structures of tetragonal SnO and PbO at 0 GPa and selected high pressures were calculated and are presented in Figure 4. SnO is a small bandgap semiconductor at ambient conditions with a measured indirect bandgap reported at approximately 0.7 eV [3,31]; PbO has a larger indirect bandgap at 1.9 eV [32]. Our calculated values of the indirect gap between Γ and M points (SnO: 0.18 eV, PbO: 1.69 V) were underestimated due to the well-known limitations of the one-electron picture in DFT [33]. In SnO, the closing of the indirect bandgap that marks the semiconductor–semi-metallic transition occurred at P = 2.57 GPa, in agreement with previous DFT calculations [9,19]. This value is lower than the experimental transition pressure observed at P = 4.3–5.1 GPa [18] and P = 4.67 GPa from an overall infrared reflectivity measurement [15].

**Figure 5:**
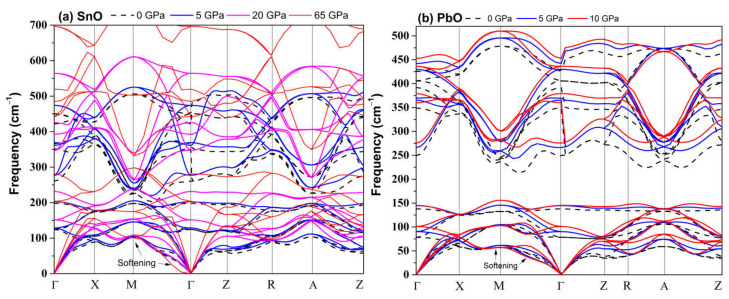
Calculated tetragonal *P4/nmm* phonon dispersion upon compression: (**a**) SnO; (**b**) PbO.

An alternative and better method to predict the pressure of the bandgap closing is to determine the bandgap’s average pressure coefficient and apply it to the experimentally measured bandgap. For SnO, we found the pressure coefficient to be −0.154 eV/GPa. Applying this value to the reported experimental indirect gap of 0.7 eV at P = 0 GPa, the pressure required to close the gap is 4.55 GPa, close to the experimental values [15]. For PbO, the average coefficient was found to be −0.057 eV/GPa. The decrease in the bandgap of PbO does not lead to a semiconductor–semi-metallic transition in the stable pressure range of 0–5 GPa. We estimate that it would occur near 50 GPa; however, compression beyond P > 5 GPa of tetragonal PbO leads to an α–β transition, which can increase the bandgap again. The calculated phonon spectra of compressed tetragonal SnO and PbO are presented in Figure 5 and exhibit only positive frequencies, indicating continued dynamical stability of this phase upon compression.

#### 3.2.2. High-Pressure Phases of SnO Predicted by Genetic Algorithm

The low enthalpy SnO phases were obtained using the GA at pressures of 5, 20, 50, and 100 GPa, and their structural properties are summarized in Table S1. The low-pressure γ-phase *Pmn2_1_* was found in the GA search to have the lowest enthalpy at 5 GPa. The GA search at 20 GPa found the *Pmmn* and *P2_1_m* structures to have lower enthalpies than the reference *P4/nmm* phase. At high pressure, the *Pbcm* phase became enthalpically favored. We calculated the difference between the formation enthalpies, *H_GA_*, of the favored SnO high-pressure phases (*Pmmn*, *Pbcm*, and octahedral *P4/nmm)* and that of the tetragonal ground state, *H_tetra_*, as a function of pressure:

Δ*H* = *H_GA_* − *H_tetra_* (2)

**Figure 6:**
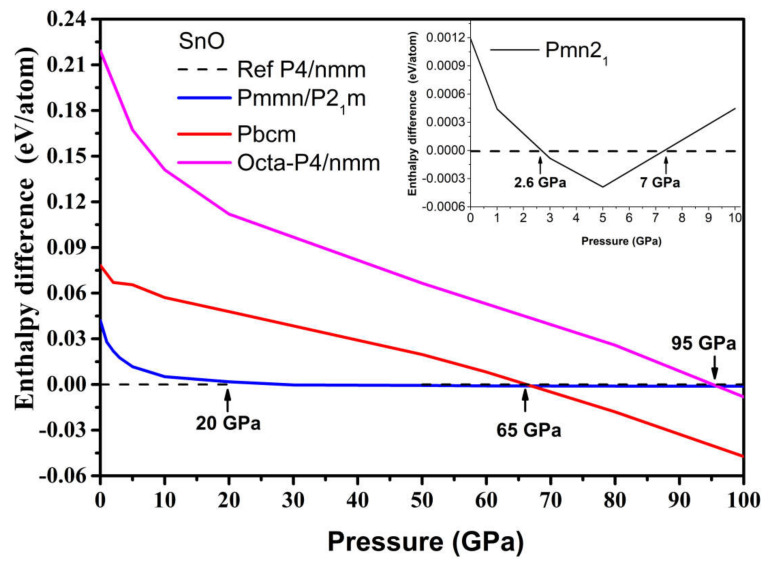
Enthalpy difference ΔH of high-pressure SnO phases, where H_GA_ is the total enthalpy of the GA phase, and H_tetra_ is the total enthalpy of the reference tetragonal SnO. Inset: enthalpy difference for the *Pmn2_1_* phase of SnO at low pressure.

The results are presented in Figure 6. The γ-phase *Pmn2_1_* became the most thermodynamically preferred phase at 2.6 GPa, albeit to a slight extent, which is in agreement with [11]. However, it became unfavorable again with respect to the tetragonal α phase at pressures above 7 GPa. The *Pmmn* orthorhombic and related distorted *P2_1_m* phases were thermodynamically stable in the pressure range between P = 20 and P = 65 GPa. This result agrees with the occurrence of monoclinic phases in experiments where P > 14 GPa [12,15]. Further increases in pressure beyond 65 GPa induced a phase transition to the *Pbcm* phase, modifying both the original pyramidal and ordered trigonal bipyramidal formations to form a disordered trigonal bipyramidal formation.

**Figure 7:**
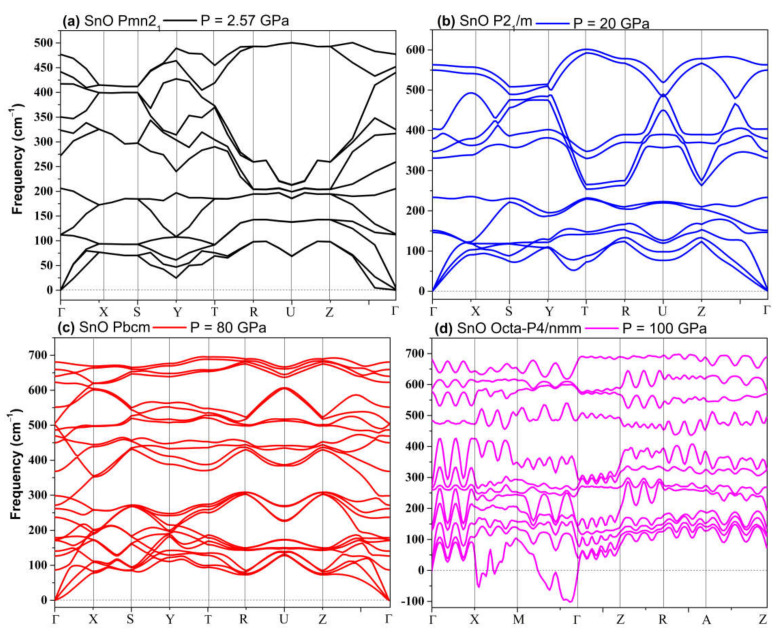
Phonon dispersion of structures of SnO: (**a**) *Pmn2_1_*; (**b**) *P2_1_/m*; (**c**) *Pbcm*; (**d**) octahedral *P4/nmm*.

The phonon spectra calculations presented in Figure 7 confirm the stability of the *Pmn2_1_*, *Pmmn*, and *Pbcm* phases at elevated pressures. Therefore, we conclude that the *Pbcm* phase of SnO is thermodynamically preferred up to 100 GPa.

#### 3.2.3. High-Pressure Phases of PbO Predicted by Genetic Algorithm

**Figure 8:**
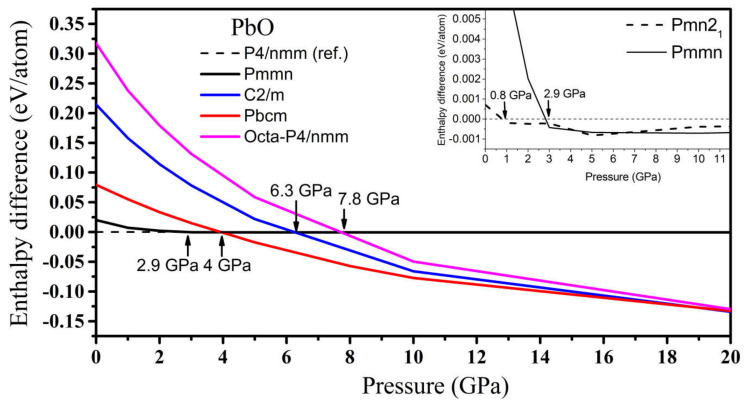
The enthalpy difference (ΔH = H_GA_ − H_tetra_) of high-pressure phases of PbO, where H_GA_ is the total enthalpy of the GA phase and H_tetra_ is the total enthalpy of the reference tetragonal PbO. Inset: the enthalpy difference for the *Pmn2_1_* phase of PbO at low pressure.

**Figure 9:**
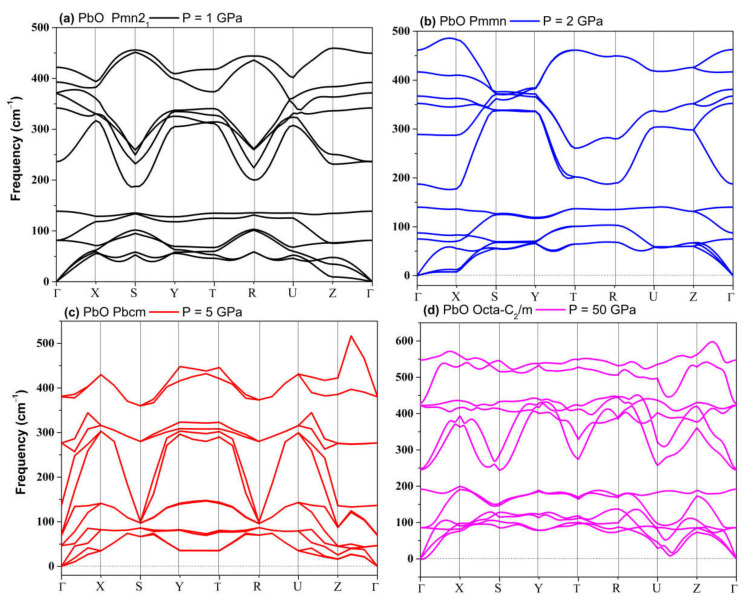
Phonon dispersion of phases of PbO: (**a**) *Pmn2_1_*; (**b**) *Pmmn*; (**c**) *Pbcm*; (**d**) Octahedral monoclinic *C_2_/m*.

In PbO, the GA identified orthorhombic *Pmn2_1_*, *Pmmn*, and *Pbcm*; octahedral *P4/nmm*; and *C_2_/m* as low enthalpy states at high pressure. Their structures are presented in Table S2. Their formation enthalpies, relative to the ambient tetragonal PbO phase, are presented in Figure 8. The γ-phase *Pmn2_1_* of PbO was energetically preferred above 0.8 GPa and in agreement with Ref. [11]. We found that the *Pmmn* phase was energetically favored above 2.9 GPa. However, upon further compression, its enthalpy remained very similar to that of tetragonal PbO (see inset Figure 8). Above 4 GPa, the β-phase *Pbcm* structure of PbO was stabilized in agreement with the α → β and γ → β transitions observed experimentally [11,16]. We also found phases with an octahedral arrangement of PbO: *C_2_/m* and *P4/nmm,* which are dynamically unstable at low pressures. All three phases of *Pbcm, C_2_/m,* and octahedral *P4/nmm* converge at P > 20 GPa both energetically and structurally (see Figure S1 for the convergence of the lattice parameters at 20 GPa). The disordered trigonal bipyramidal *Pbcm* structure will gradually reconstruct into the more symmetric octahedral *C_2_/m* formation upon compression. The phonon dispersion curves were calculated for all phases and are presented in Figure 9. These results confirm the dynamical stability of the octahedral monoclinic *C_2_/m* PbO at P = 50 GPa as well as the γ-*Pmn2_1_*, *Pmmn*, and β-*Pbcm* phases of PbO at their transition pressures.

## 4. Discussion

### 4.1. Structures of SnO and PbO under Pressure—Summarizing Our Results and Their Validity and Comparing Them to Previous Theoretical and Experimental Results

**Figure 10:**
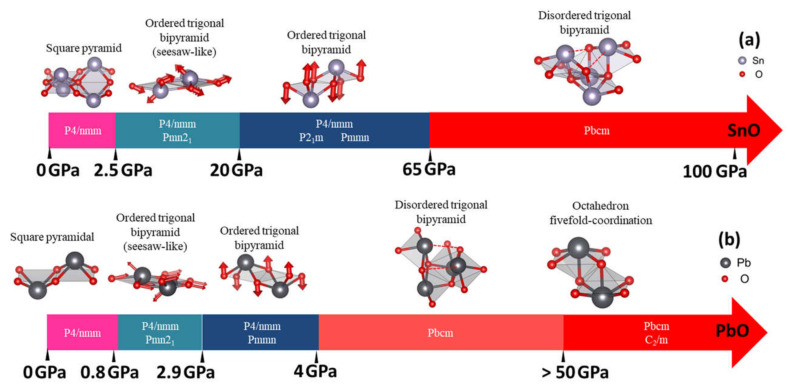
Summary of the structural evolution in pressure-induced transitions of SnO/PbO. The red arrows indicate the shifted position of the Sn/Pb and O atoms in ordered trigonal pyramidal arrangements compared to the square pyramidal arrangement. The enthalpy differences in pressure ranges with multiple phases are insignificant (less than 5 meV) between the phases: (**a**) The structural evolution in pressure-induced transitions of SnO; (**b**) The structural evolution in pressure-induced transitions of PbO.

In Figure 10, we summarize our results for the stable phases of SnO and PbO as a function of pressure. Under ambient conditions, our results reveal the existence of monoclinic *P2_1_/m* SnO and orthorhombic *Pmmn* PbO as well as tetragonal *P4/nmm*. These new phases may be obtained through the decompression process from the high-pressure phases. In addition, compression introduces the possibility of obtaining orthorhombic γ-phase *Pmn2_1_,* which is unstable under ambient conditions. This phase is a slight distortion of the tetragonal pyramidal arrangement via shear stress effect, as suggested by previous experiments [12,15,16]; the effect of shear stress σ_xz_ can trigger the motion of both Sn and O atoms in y and z directions to produce a trigonal bipyramidal γ phase. Figure 10a (range 2.5–10 GPa) and 10b (range 0.8–2.9 GPa) describe the atomic shift of Sn and O atoms to distort *P4/nmm* structure into the γ-phase. Meanwhile, monoclinic *P2_1_/m* and orthorhombic *Pmmn* can be obtained by the stresses σ_xx_ and σ_xy_ (σ_zz_ does not transform the tetragonal structure). This distortion is related to the shifting of a couple of O atoms in opposite directions along the z-direction. This shifting of O atoms from the original plane of *P4/nmm* is visualized in Figure 10a (SnO: range 20–65 GPa) and 10b (PbO: range 2.9–4 GPa).

In the calculation, the optimized γ-SnO structure, refined at 2.57 GPa, presents a splitting of 0.42% between the lattice parameters *a* and *b*, which is very close to the value of 0.58% measured experimentally [11] and the 0.29% value found in a previous DFT calculation [14]. In contrast γ-PbO has a splitting of 2.4%, which is less than the experimental values of 7.2% [11] and 9.8% [16]. At a higher pressure, the preference for the monoclinic *P2_1_/m* SnO over γ-SnO corresponds to the results obtained from experimental compression at 17.5 GPa, using MgO as the pressure medium [15]. Our optimized structures (at the same pressure) have a splitting *b/a* ratio equal to 1.39% with β = 90.16°, compared with a splitting *b/a* ratio of 0.64% and β = 90.25° in the experimental data.

Upon additional compression, the disordered trigonal bipyramidal *Pbcm* phase of PbO found in our study at 4 GPa agrees with experimental reports [11,16,34]. In [34], the variation of *Pbcm* PbO *a*- and *b*-lattice parameters (equivalent to *b* and *c* in [34]) during compression indicated that the *Pbcm* structure gradually transforms into an ordered octahedral structure. This suggests that the ordered octahedral structure can manifest when the Pb atom approaches an adjacent O (dashed line in Figure 10) and forms a new Pb–O bond for the trigonal bipyramidal *Pbcm.* The phonon dispersion calculations for both SnO and PbO implied that this octahedral phases could be hard to stabilize due to the unstable phonon modes. Our newfound octahedral monoclinic *C_2_/m* phase of PbO required compression up to 50 GPa to stabilize all phonon modes, and the octahedral *P4/nmm* phase of SnO remains unstable up to the limit of our study at 100 GPa. Meanwhile, the newfound phase *Pbcm* of SnO has not yet been reported in the literature, but the enthalpy and phonon calculation indicate its stability at P > 65 GPa.

### 4.2. Elastic Instability of SnO/PbO upon Compression

**Figure 11:**
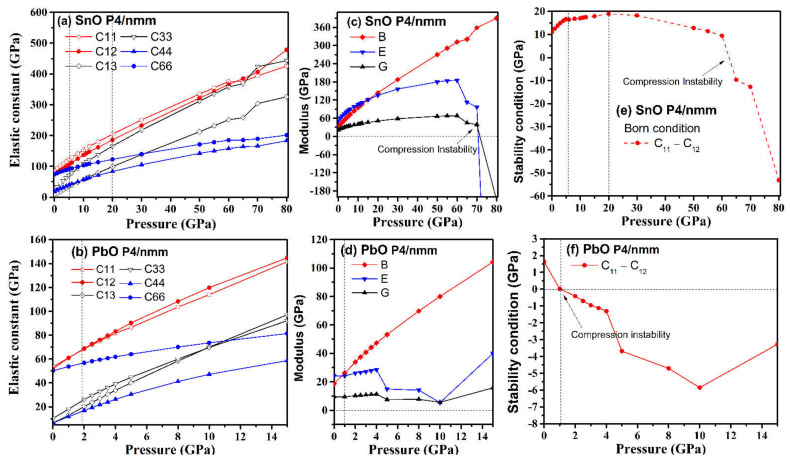
Comparison of *P4/nmm* SnO/PbO elastic properties upon compression: (**a**,**b**) elastic constants C_ij_; (**c**,**d**) bulk (B), Young (E), and shear (G) modulus; (**e**,**f**) validation of the Born criterion: C_11_–C_12_ > 0.

Elastic constant coefficients of mechanically stable tetragonal structures must satisfy the Born stability condition [35]: C_11_ − C_12_ > 0, C_33_ (C_11_ + C_12_) − 2C_13_^2^ > 0, C_44_ > 0, C_66_ > 0. We have calculated the elastic properties of tetragonal *P4/nmm* SnO and PbO under compression, and the results are presented in Figure 11.

In general, the elastic constants increased with pressure. However, the combinations of elastic constants that form Young’s modulus and the shear modulus did not behave monotonously with pressure, peaking at approximately 60 GPa for SnO and 4 GPa for PbO. The Born stability condition C_11_ − C_12_ > 0 was violated by SnO at 63 GPa and by PbO at 1 GPa, with another significant drop occurring later, at 4 GPa. The mechanical instability of PbO at a low pressure of 1 GPa corresponded to the phase transition to γ-phase found above in Figure 8. The instability upon compression of the tetragonal structure at 65 GPa for SnO and 4 GPa for PbO corresponded with the *Pbcm* phase transitions. However, this was not the thermodynamic driving force for SnO. In the low-pressure regime, the tetragonal structures of both SnO and PbO were susceptible to shear stress due to the small difference between the C_11_ and C_12_ elastic coefficients. This was particularly true of tetragonal PbO, as the value of C_11_–C_12_ decreased close to 0 GPa, whereas, in SnO, the value of C_11_–C_12_ increased at first before dropping rapidly at a pressure above 20 GPa. This suggests that a small shear stress can induce transitions of these layered structures at low pressure.

The elastic properties of orthorhombic *Pmn2_1_*, *Pmmn*, and *Pbcm* of SnO/PbO were calculated at high pressures, and the results are presented in Table 3. They indicate that the orthorhombic phases at high pressure satisfy the Born stability conditions [35]: C_ii_ > 0, C_ii_ + C_jj_ − 2C_ij_ > 0, C_11_ + C_22_ + C_33_ + 2(C_12_ + C_13_ + C_23_) > 0. The necessary and sufficient Born criteria for orthorhombic structure, as proposed in [36], are also validated: C_11_C_22_ > C_12_^2^, C_11_C_22_C_33_ + 2C_12_C_13_C_23_ − C_11_C_23_^2^ − C_22_C_13_^2^ − C_33_C_12_^2^ > 0. At a high pressure, the elastic stability of the octahedral *P4/nmm* PbO is confirmed, as all the Born criteria (C_11_ − C_12_ > 0, C_33_ (C_11_ + C_12_) − 2C_13_^2^ > 0, C_44_ > 0, C_66_ > 0) are satisfied.

### 4.3. Electronic Structure and the Role of Lone Pairs upon Compression

**Figure 12:**
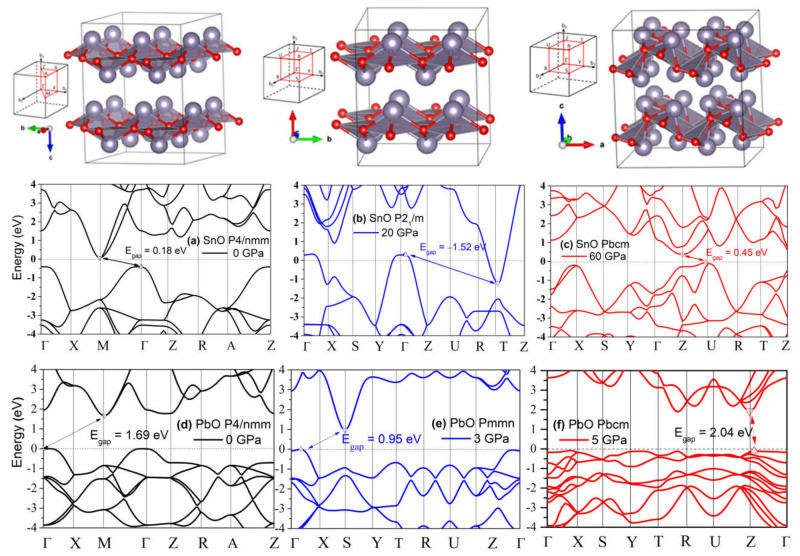
The polymorphs of layered SnO/PbO and their electronic band modification upon compression. The gaps between valence band maximum (VBM) and conduction band minimum (CBM) are marked by arrows and values. A negative gap value indicates an overlap of CBM and VBM due to the reduced distance between the two layers: (**a**) Band structure of *P4/nmm* SnO at 0 GPa; (**b**) Band structure of *P2_1_/m* SnO at 20 GPa; (**c**) Band structure of *Pbcm* SnO at 60 GPa; (**d**) Band structure of *P4/nmm* PbO at 0 GPa; (**e**) Band structure of *Pmmn* PbO at 3 GPa; (**f**) Band structure of *Pbcm* PbO at 5 GPa.

The compression of both SnO and PbO reduces the bandgap. As discussed in the context of Figure 4, the semiconductor–semi-metallic transition occurs at P = 2.57 GPa in SnO *P4/nmm*, whereas this transition does not occur in PbO at a pressure up to 5 GPa. The transitions of square pyramidal SnO *P4/nmm* to the trigonal bipyramidal and subsequently to the octahedral *Pbcm* structure correspond with modifications in the electronic structure that trigger relocation of the VBM from Γ to a nearby point. In Figure 12b,e, the band structures of SnO *P2_1_/m* and PbO *Pmmn* present this VBM relocation with limited modification to the electronic structure. More significant modification happened upon further compression, with the electronic structures of SnO and PbO in *Pbcm* increasing the bandgap. Specifically, the overlapping bandgaps of the *P4/nmm* and *P2_1_/m* phases of SnO are replaced by an indirect bandgap at Z–U in the *Pbcm* phase (Figure 12c). A similar reopening of the PbO bandgap to 2.04 eV in the vicinity of the Z point is also observed in Figure 12d–f. The reduction and subsequent reopening of the PbO bandgap was reported in the α → β and γ → β transitions in experiments [11,34]. The pressure-induced variation in the lower conducting and upper valence bands is next discussed via the lone pairs model of SnO/PbO.

**Figure 13:**
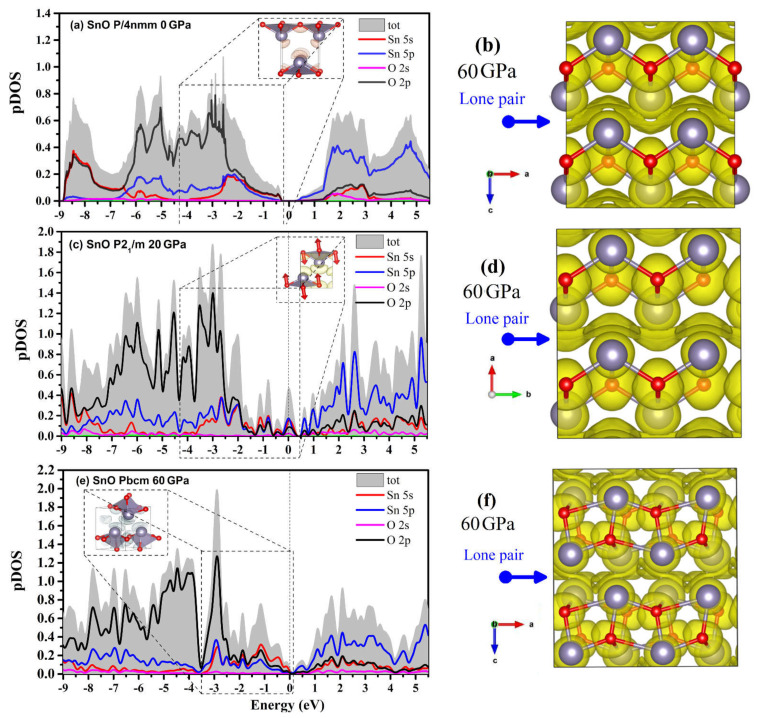
pDOS and the lone pairs of three polymorphs of SnO. The marked region indicates the hybridization of the Sn–O states of lone pairs via ILDOS. The electron localization function for a supercell of two layers of each structure is calculated at 60 GPa. The ELF and ILDOS iso-surface levels are set at 0.15 e/Å^3^ and 0.006 e/Å^3^, respectively: (**a**) pDOS and ILDOS of *P4/nmm* SnO at 0 GPa; (**b**) ELF of *P4/nmm* SnO at 60 GPa; (**c**) pDOS and ILDOS of *P2_1_/m* SnO at 20 GPa; (**d**) ELF of *P2_1_/m* SnO at 60 GPa; (**e**) pDOS and ILDOS of *Pbcm* SnO at 60 GPa; (**f**) ELF of *Pbcm* SnO at 60 GPa.

The projected DOS (pDOS) of the SnO phases and a visualization of the lone pairs from the integrated local DOS (ILDOS) and electron localization function (ELF) are presented in Figure 13. The dome shape of the lone pairs in *P4/nmm* at 0 GPa (formed by hybridization between Sn 5s, 5p with the O 2p states) can be seen in the ILDOS in Figure 13a. When compressed to 60 GPa, the *P4/nmm* ELF shows that the lone pairs between two layers of SnO assemble into a layer due to the reduction in the intralayer distance along the c axis. The compression of the lone pairs appears as the smearing of pDOS continuously fills the Fermi level. The contribution of the Sn 5s states at the Fermi level is increased in the *P2_1_/m* structure (Figure 13c). The rise in Sn 5s states is correlated with the VBM relocation in Figure 12b. The ELF calculated for the SnO *P2_1_/m* structure also shows a similarly smeared lone pair layer, as in *P4/nmm*. At 60 GPa, the pDOS shows that the *Pbcm* lone pairs layer contains two contributions: a hybridization of an O 2p state with weaker Sn 5s and 5p contributions, and a Sn 5s state hybridized weakly with O 2p and Sn 5p states. Thus, the modification of VBM and CBM of SnO *Pbcm* in Figure 12c coincides with the separation of the Sn 5s and O 2p state contributions to the DOS in Figure 13e. In the *Pbcm* phase, the lone pairs rotate to mediate not only the interlayer interaction but also the intralayer Sn–Sn interaction.

The transition between different layered structures of SnO/PbO can be presumed as follows: in *P4/nmm*, the lone pairs act as a dome on top of the Sn that reduces interaction between the direct interlayer Sn atoms. The effect of the dome-shaped lone pair of Sn/Pb is gradually smeared and diminished upon compression. This more isotropic layer may act as a “lubricant” and reduce the resistance to shear so that the layers of SnO can easily slide over each other. When two SnO/PbO adjacent layers slide, O atoms at the two main sites in the O plane of the square pyramidal structure move in two opposite directions. These O motions induce the splitting of the a/b lattice into the orthorhombic structure. The *Pbcm* phase results from continued shear of the two layers as the lone pairs are twisted from the apex to the side of the square pyramidal structure. Like the lone pairs of the trigonal bipyramidal *P2_1_/m* at 20 GPa, those of *Pbcm* at 60 GPa can also be considered a lubricant between the two layers. In Figure 13f, the asymmetric lone pairs of the *Pbcm* phase allow the Sn atoms to shift to the side unblocked by the lone pairs. This explains why the two layers can easily slide to form the octahedral monoclinic *C_2_/m* in PbO or octahedral *P4/nmm* in SnO.

**Figure 14:**
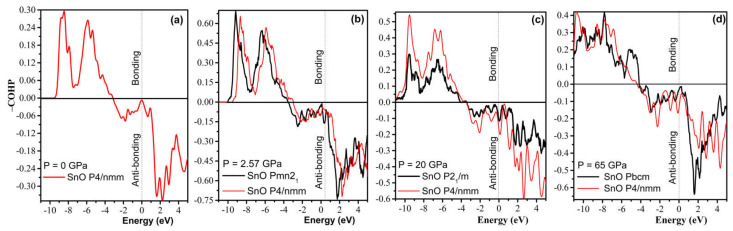
Calculated −COHP diagram of the high-pressure polymorphs of SnO compared to the reference tetragonal *P4/nmm* at different pressures. Positive energy values of −COHP bonding illustrate the bonding regions, whereas negative energy values describe the antibonding regions: (**a**) *P4/nmm*; (**b**) *Pmn2_1_* vs. *P4/nmm*; (**c**) *P2_1_/m* vs. *P4/nmm*; (**d**) *Pbcm* vs. *P4/nmm*.

Further analysis of chemical bonding by way of a COHP calculation in Figure 14 reveals that the compression of the *P4/nmm* phase slowly increases the antibonding contribution. Hence, a shear to form the orthorhombic–monoclinic distortion can be triggered at a low pressure to reduce unfavorable antibonding. Further high-pressure compression significantly increases instability, as the Fermi level is pushed deeper into the upper antibonding region. Thus, the transformation to *Pbcm* is necessary to reduce the antibonding contribution. As suggested by a revised model of lone pairs by Walsh et al. [37], the formation of stereochemically active lone pairs in SnO/PbO is sensitive to the relative interaction of cation s and anion p states. The compression of the *P4/nmm* structure increases the contribution of the Sn/Pb s states in the antibonding part while reducing the contribution of the Sn/Pb p states in the bonding, which results in the electronic instability found by the COHP analysis.

## 5. Conclusions

We predicted the high-pressure phases of SnO/PbO and their metastable phases under ambient conditions and at high pressures using a global search with a genetic algorithm (GA). We were able to explore the pressure-induced transitions fully: (i) the metastable phase of SnO at 0 GPa is the monoclinic *P2_1_m* structure and for PbO theorthorhombic *Pmmn* structure. Both these phases are slightly distorted from the tetragonal structure and can be obtained by decompressing back from the high-pressure phase. (ii) Under pressure, the orthorhombic and monoclinic phases become more favorable than the tetragonal phase. At low pressure, the phase transitions of α → γ *(Pmn2_1_*) for SnO at 2.5 GPa and PbO at 0.8 GPa are in good agreement with the experimental results. Further transitions to the ordered trigonal bipyramidal structures of the monoclinic *P2_1_/m* phase of SnO and the *Pmmn* phase of PbO occur at a higher pressure than the γ phase. (iii) Both the square pyramidal *P4/nmm* and ordered trigonal bipyramidal (γ phase, *P2_1_m* SnO, and *Pmmn* PbO) structures transform into the *Pbcm* phases upon further compression. (iv) Our calculations also predict that the monoclinic *C_2_/m* representing an ordered octahedral formation can be obtained when P > 50 GPa for PbO. The stable *Pbcm* structure of SnO at 65 GPa and the monoclinic *C_2_/m* structure of PbO at 50 GPa, found in our calculations, have not yet been reported in the literature.

The mechanism of the high-pressure phase transitions is explained by elastic instability and the behavior of the lone pairs: (i) The orthorhombic–monoclinic transitions in the high-pressure phase are associated with elastic instability. PbO is more susceptible to breaking tetragonal symmetry to form an orthorhombic structure than SnO. At a high enough pressure, this shear drives the breaking of the square pyramidal formation of *P4/nmm* to form the trigonal bipyramidal structure first and then the disordered arrangement of *Pbcm*. (ii) The sliding of the SnO/PbO layers originates from weakening the lone pairs upon compression. The smeared lone pairs in Sn/Pb act as a lubricant layer so that the SnO/PbO layers can shift over each other. This shear into an orthorhombic–monoclinic structure strengthens the bonding and reduces the antibonding instability at the Fermi level upon compression. (iii) The band gap modulation induced by the structural transition suggests a semiconductor–metal–semiconductor sequence of transitions as pressure is increased.

Finally, the present study illustrates the utility of combining first-principles DFT modeling with evolutionary algorithms to study the complex phase landscape of layered and other structured materials. It is seen that our predicted structures are in good agreement with the wide variety of structures observed experimentally. Consequently, we expect this strategy to be widely applied in the future, e.g., to analyze the complex pressure-induced transitions in other layered structures.

## Figures and Tables

**Table 1:** Low enthalpy SnO phases predicted by the GA at P = 0 GPa and their structural parameters (Sn: light blue and O: red).

Phase	E_f_ (eV/atom)	Structure	Lattice Parameters (Å)	No. of Atoms Unit Cell	Spacegroup (SG) and Wyckoff Atomic Position
*P4nmm* (Tetragonal)	0	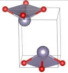	a = b = 3.8699 c = 5.0426	4	SG = 129 Sn 2c 0.25 0.25 0.2296 O 2a 0.75 0.25 0.0
*P2_1_m* (Monoclinic)	0.0019	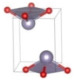	a = 3.9128 b = 3.8225 c = 4.9846 β = 90.339°	4	SG = 11 Sn 2e 0.7515 0.25 0.2316 O 2e 0.2499 0.25 0.0045
*Pmmn* (Orthorhombic)	0.0043	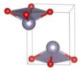	a = 3.7826 b = 3.9520 c = 4.9417	4	SG = 59 Sn 2b 0.25 0.75 0.2333 O 2a 0.25 0.25 0.0081
*Pbcm* (Orthorhombic)	0.0485	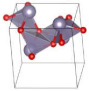	a = 6.0846 b = 5.6618 c = 4.5270	8	SG = 57 Sn 4d 0.7179 0.4774 0.25 O 4d 0.6161 0.1068 0.25
*P2_1_/c* (Monoclinic)	0.0583	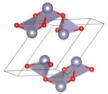	a = 6.0443 b = 4.2685 c = 6.3114 β = 109.3927°	8	SG = 14 Sn 4e 0.7572 −0.0025 0.7682 O 4e 0.8826 0.1828 0.0965
*P2_1_3* (Cubic)	0.084	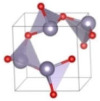	a = b = c = 5.3448	8	SG = 198 Sn 4a 0.4891 0.4891 0.4891 O 4a 0.8809 0.8809 0.8809
*P4/nmm* (Tetragonal)	0.151	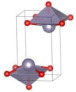	a = b = 3.4079 c = 6.2247	4	SG = 198 Sn 2c 0.25 0.25 0.2199 O 2c 0.25 0.25 0.8758

**Table 2:** Low enthalpy PbO phases predicted by GA at P = 0 GPa and their structural parameters (Pb: gray and O: red).

Phase	E_f_ (eV/Atom)	Structure	Lattice Parameters (Å)	No. of Atoms Unit Cell	Spacegroup (SG) and Wyckoff Atomic Position
*P4nmm* (Tetragonal)	0	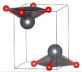	a = b = 4.0707 c = 5.4175	4	SG = 129 Pb 2c 0.25 0.25 0.2186 O 2a 0.75 0.25 0.0
*Pmmn* (Orthorhombic)	0.0009	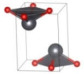	a = 3.9670 b = 4.1654 c = 5.5171	4	SG = 59 Pb 2a 0.25 0.25 0.7851 O 2b 0.25 0.75 −0.0096
*P42_1_m* (Tetragonal)	0.042	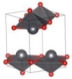	a = b = 5.134 c = 6.4102	8	SG = 113 Pb 4e 0.7588 0.2588 0.7867 O 4e 0.7145 0.2145 0.1348
*C2* (Monoclinic)	0.043	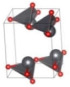	a = 4.9607 b = 5.2977 c = 6.4035 β = 91.995°	8	SG = 5 Pb 4c 0.7414 0.0642 0.7849 O 4c 0.2437 0.1338 0.8664
*Pmn2_1_* (Orthorhombic)	0.044	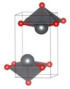	a = 3.5834 b = 6.2899 c = 3.7034	4	SG = 31 Pb 2a 0.6425 0.6421 0.1415 O 2a 0.7560 0.7437 0.7562
*P2_1_3* (Cubic)	0.046	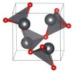	a = b = c = 5.522	8	SG = 198 Pb 4a 0.2437 0.2437 0.2437 O 4a 0.6420 0.6420 0.6420
*P4_2_* (Tetragonal)	0.0998	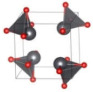	a = b = 5.5937 c = 5.2962	8	SG = 77 Pb 4d 0.7702 0.7763 0.2180 O 4d 0.8184 0.1902 0.2811

**Table 3:** Elastic constants C_ij_ (GPa), Voigt–Reuss–Hill average bulk modulus B (GPa), Voigt–Reuss–Hill average shear modulus G (GPa), Young’s modulus E (GPa), and Poisson’s ratio ν of the orthorhombic and octahedral phases of SnO and PbO.

**SnO**	**C_11_**	**C_12_**	**C_13_**	**C_22_**	**C_23_**	**C_33_**	**C_44_**	**C_55_**	**C_66_**	**B**	**G**	**E**	**ν**
*Pmn2_1_* 2.5 GPa	114.6	18.0	78.5	58.0	27.1	112.7	34.9	84.1	34.7	52.6	36.1	88.0	0.221
*Pmmn* 20 GPa	167.2	101.5	103.6	207.4	189.6	203.5	123.6	83.8	82.7	146.5	49.1	130.5	0.330
*Pbcm* 80 GPa	241.8	143.2	113.5	585.8	252.3	396.7	85.6	85.6	132.8	224.7	94.5	248.6	0.316
**PbO**	**C_11_**	**C_12_**	**C_13_**	**C_22_**	**C_23_**	**C_33_**	**C_44_**	**C_55_**	**C_66_**	**B**	**G**	**E**	**ν**
*Pmn2_1_* 1 GPa	20.5	13.8	15.7	73.9	45.5	75.1	53.5	17.5	16.2	27.9	19.8	47.9	0.209
*Pmmn* 2 GPa	88.8	77.3	32.9	91.8	38.3	35.3	28.5	25.7	63.9	45.6	21.1	54.8	0.298
*Pbcm* 5 GPa	161.1	49.1	43.0	90.8	26.2	84.5	11.4	18.9	42.3	59.8	67.4	25.6	0.311
*P4/nmm* 50 GPa	355.3	196.9	241.6	-	-	435.7	59.5	-	167.9	268.2	83.6	226.9	0.358

## Data Availability

The data that support the findings of this study are available from the corresponding author upon reasonable request.

## Supplementary Materials

The following are available online at https://www.mdpi.com/article/10.3390/ma14216552/s1, Table S1: Low enthalpy SnO phases predicted by GA at 5, 20, 50, and 100 GPa and their structural parameters, Table S2: Low enthalpy PbO phases predicted by GA at 1, 5, 20, and 50 GPa and their structural parameters, Figure S1: Structural variation of the low- and high-pressure PbO and SnO phases upon compression.
