# Supplementary material for: High-Pressure Phases of SnO and PbO: A Density Functional Theory Combined with an Evolutionary Algorithm Approach
Source: Materials (Basel). 2021 Nov 1;14(21):6552. doi: 10.3390/ma14216552 (PMC8585256; doi:10.3390/ma14216552)
Supplement: Supplementary file 1 [file materials-14-06552-s001.zip › materials-1397573-supplementary.pdf]

Supplementary Materials

High-Pressure Phases of SnO and PbO: A Density Functional Theory Combined with an Evolutionary Algorithm Approach

Long Truong Nguyen and Guy Makov *

Department of Materials Engineering, Faculty of Engineering Sciences, Ben-Gurion University of the Negev, Beer-Sheva 84105, Israel; nguyenl@post.bgu.ac.il
* Correspondence: makovg@bgu.ac.il

Citation: Nguyen, L.T.; Makov, G. High-Pressure Phases of SnO and PbO: A Density Functional Theory Combined with an Evolutionary Algorithm Approach. *Materials* **2021**, *14*, 6552. https://doi.org/10.3390/ma14216552

Academic Editors: Quanjun Li, Xiaobing Liu and Jose Antonio Alonso

Received: 11 September 2021
Accepted: 28 October 2021
Published: 1 November 2021

Publisher's Note: MDPI stays neutral with regard to jurisdictional claims in published maps and institutional affiliations.

Copyright: © 2021 by the authors. Licensee MDPI, Basel, Switzerland. This article is an open access article distributed under the terms and conditions of the Creative Commons Attribution (CC BY) license (http://creativecommons.org/licenses/by/4.0/).

Details about the refined structure of the low enthalpy SnO phases at each global optimization GA step of 5, 20, 50, and 100 GPa are shown in Table S1. In a low-pressure search, P = 5 GPa and the γ-phase transition is found at 2.6 GPa, as suggested by Adams [11]; however, the global optimization search did not spot this phase at P > 5 GPa. In the P = 20 GPa search, the *Pmmn* and *$P2_1m$* structures were discovered, with the splitting ratios of b and a being 1.06% and 0.49%, respectively. The P = 50 GPa and P = 100 GPa searches by GA revealed the *Pbcm* phase and its analogs of the octahedral structure, which are less favored at 0 GPa. The octahedral structure was very favorable in the global search at P = 100 GPa, as all the generations converged to these phases.

Table S1. Low enthalpy SnO phases predicted by GA at 5, 20, 50, and 100 GPa and their structural parameters. Reference tetragonal SnO, compressed from experimental input at the same pressure point, is included for comparison.

Pressure	Preferred phase	Formation energy (eV/atom)	Lattice parameters (Å)	Wyckoff atomic position
5 GPa	*P4/nmm* (ref.)	0	a = b = 3.800<br>c = 4.557	Sn 0.0 0.5 0. 2538<br>O 0.0 0.0 0.0
	$P2_1/m$	−0.00024	a = 3.792, b = 3.806<br>c = 4.559<br>β = 90.028°	Sn 0.7502 0.25 0.2539<br>O 0.2500 0.25 −0.0008
	γ phase<br>$Pmn2_1$	−0.00041	a = 3.788, b = 3.809<br>c = 4.557	Sn −0.0106 0.0 0.2460<br>O 0.4896 0.0 0.5013
20 GPa	*P4/nmm* (ref.)	0	a = b = 3.6576<br>c = 4.1730	Sn 0.0 0.5 0. 2796<br>O 0.0 0.0 0.0
	Pbcm	0.047	a = 4.2784, b = 4.9704<br>c = 5.2077	Sn 0.25 0.2614 0.5048<br>O 0.25 0.1368 0.0952
	$P2_1m$	−0.0011	a = 3.6864, b = 3.7044<br>c = 4.1199<br>β = 90.073°	Sn 0.75002 0.25 0.7774<br>O 0.24998 0.25 0.5011
	Pmmn	−0.0018	a = 3.6322, b = 3.6706<br>c = 4.1769	Sn 0.25 0.25 0.7800<br>O 0.25 0.75 0.4977
50 GPa	*P4/nmm* (ref.)	0	a = b = 3.5079<br>c = 3.8962	Sn 0.0 0.5 0. 2984<br>O 0.0 0.0 0.0
	Pbcm	0.0058	a = 4.1138, b = 4.6283<br>c = 4.9637	Sn 0.25 −0.0141 0.7758<br>O 0.25 0.4124 0.6362
	Pmmn	−0.00062	a = 3.4930, b = 3.5147<br>c = 3.9043	Sn 0.25 0.25 0.7015<br>O 0.25 0.75 0.0011
100 GPa	*P4/nmm* (ref.)	0	a = b = 3.3799<br>c = 3.6646	Sn 0.0 0.5 0. 3122<br>O 0.0 0.0 0.0
	P4/nmm<br>Octahedral	−0.0082	a = b = 2.8728<br>c = 4.9304	Sn 0.25 0.25 0.1288<br>O 0.25 0.25 0.7126
	$P2_1/m$<br>Octahedral	−0.0081	a = 2.8726, b = 2.8730<br>c = 4.9300<br>β = 90.024°	Pb 0.7501 0.25 0.1288<br>O 0.2499 0.25 0.7126
	Pbcm	−0.047	a = 3.955, b = 4.338<br>c = 4.7620	Sn 0.25 −0.0203 0.7861<br>O 0.25 0.4190 0.6335

Table S2 summarizes the metastable phases of the preferred PbO phases at each global optimization GA step at 1, 5, 20, and 50 GPa. Similar to SnO, the γ-phase *$Pmn2_1$* was found by the search at 1 GPa but not at a higher pressure. Meanwhile, the orthorhombic *Pmmn* was found by the searches at 0, 1, and 5 GPa. This phase was also stable at a low pressure and 0 GPa, whereas the γ phase is unstable at 0 GPa. The searches at 5 and 20 GPa reveal several transition paths: the square pyramidal and ordered trigonal bipyramidal phases were replaced by the disordered trigonal bipyramidal *Pbcm* and *$P2_1/m$* and

then the octahedral C_2/m. Upon further searching at 50 GPa, we reached a similar convergence with SnO to the octahedral phase.

Table S2. Low enthalpy PbO phases predicted by GA at 1, 5, 20, and 50 GPa and their structural parameters. Reference tetragonal PbO, which is compressed from experimental input at the same pressure point, is included for comparison.

Pressure	Preferred phase	Formation energy (eV/atom)	Lattice parameters (Å)	Wyckoff atomic position
1 GPa	*P4/nmm* (ref.)	0	a = b = 4.0547<br>c = 5.1452	Pb 0.0 0.5 0. 2303<br>O 0.0 0.0 0.0
	Pmmn	0.00004	a = 4.000, b = 4.1125<br>c = 5.1370	Pb 0.75 0.25 0.7695<br>O 0.25 0.25 −0.0062
	γ-phase<br>$Pmn2_1$	−0.0001	a = 4.0356, b = 4.1187<br>c = 4.8936	Pb 0.0 −0.0116 0.7389<br>O 0.0 0.4886 0.5053
5 GPa	*P4/nmm* (ref.)	0	a = b = 4.0269<br>c = 4.5695	Pb 0.0 0.5 0.2569<br>O 0.0 0.0 0.0
	Pmmn	−0.0001	a = 3.9752, b = 4.0800<br>c = 4.5693	Pb 0.75 0.25 0.2432<br>O 0.25 0.25 0.4934
	β-phase<br>*Pbcm*	0.0051	a = 4.9334, b = 4.9397<br>c = 5.7648	Sn 0.25 −0.0002 0.7422<br>O 0.25 0.0083 0.3509
20 GPa	*P4/nmm* (ref.)	0	a = b = 4.0410<br>c = 3.7151	Pb 0.0 0.5 0.3007<br>O 0.0 0.0 0.0
	Pbcm	−0.016	a = 4.7228, b = 4.7269<br>c = 5.3931	Pb 0.25 0.5017 0.7625<br>O 0.25 0.5077 0.3489
	$P2_1/m$<br>Octahedral	−0.017	a = 3.3411, b = 3.3426<br>c = 5.3966<br>β = 90.057°	Pb 0.2505 0.25 0.2624<br>O 0.2497 0.25 0.8491
	C_2/m	−0.017	a = b = 4.7278<br>c = 5.3932<br>β = 90.068°	Pb 0.7496 0.0 0.2376<br>O 0.2497 0.0 0.3487
50 GPa	*P4/nmm* (ref.)	0	a = b = 4.0025<br>c = 3.2096	Pb 0.0 0.5 0.3385<br>O 0.0 0.0 0.0
	P4/nmm<br>Octahedral	−0.025	a = b = 3.1672<br>c = 5.1609	Pb 0.25 0.25 0.6459<br>O 0.25 0.25 0.2232
	$P2_1/m$<br>Octahedral	−0.025	a = 3.1666, b = 3.1667<br>c = 5.1615<br>β = 90.058°	Pb 0.2501 0.25 0.2768<br>O 0.2499 0.25 0.8542

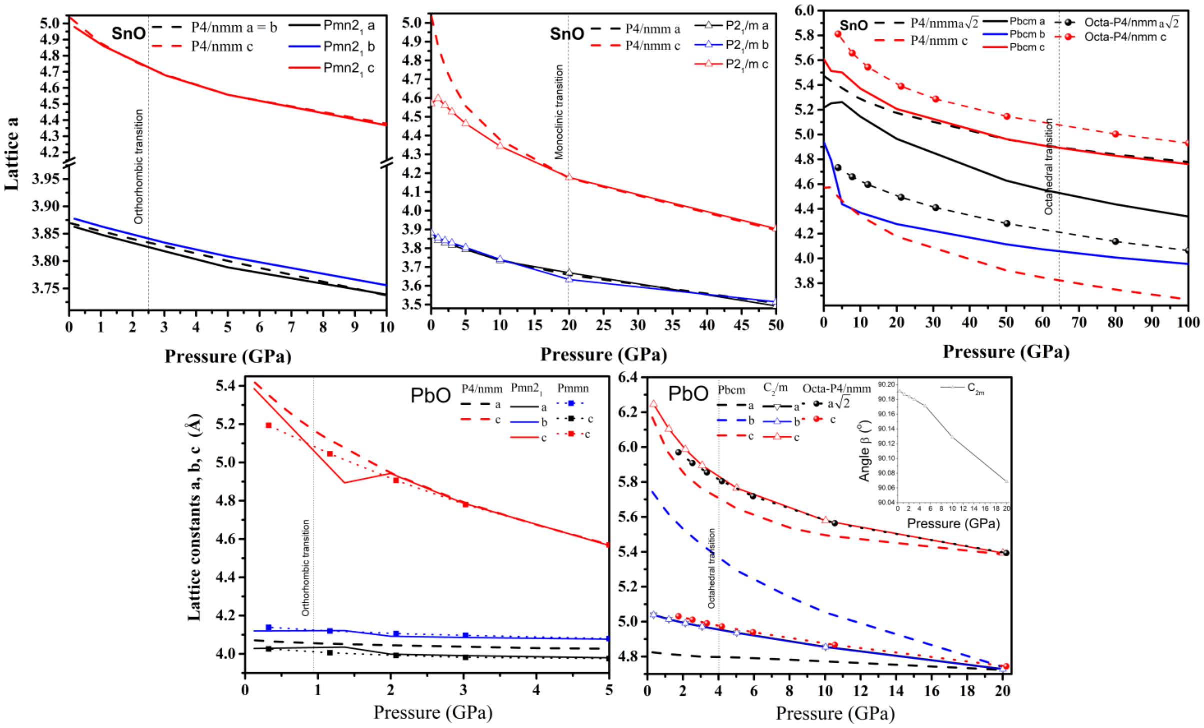

Figure S1. Structural variation of the low- and high-pressure PbO and SnO phases upon compression.
